# Utilization of hematopoietic cell transplantation and cellular therapy technology in Europe and associated Countries. Using the 2022 activity survey data to correlate with economic and demographic factors. A report from the EBMT

**DOI:** 10.1038/s41409-024-02459-0

**Published:** 2024-11-22

**Authors:** Jakob R. Passweg, Helen Baldomero, Tobias Alexander, Emanuele Angelucci, Dina Averbuch, Ali Bazarbachi, Fabio Ciceri, Greco Raffaella, Mette D. Hazenberg, Krzysztof Kalwak, Donal P. McLornan, Antonio M. Risitano, Annalisa Ruggeri, John A. Snowden, Anna Sureda

**Affiliations:** 1 Hematology Division, EBMT Activity Survey Office, University Hospital, Basel, Switzerland; 2 Department of Rheumatology and Clinical Immunology, Charité - Universitätsmedizin Berlin, Berlin, Germany; 3 Hematology and Cellular Therapy Unit. IRCCS Ospedale Policlinico San Martino, Genova, Italy; 4 Faculty of Medicine, Hebrew University of Jerusalem; Hadassah Medical Center, Jerusalem, Israel; 5 Department of Internal Medicine, Bone Marrow Transplantation Program, American University of Beirut, Beirut, Lebanon; 6 Unit of Hematology and Bone Marrow Transplantation, IRCCS San Raffaele Hospital, Vita-Salute San Raffaele University, Milan, Italy; 7 Department of Hematology, Amsterdam University Medical Centres, University of Amsterdam, and Department of Hematopoiesis, Sanquin Research, Amsterdam, The Netherlands; 8 Clinical Department of Pediatric BMT, Hematology and Oncology, Wroclaw Medical University, Wroclaw, Poland; 9 Department of Haematology, University College London Hospitals NHS Foundation Trust, London, UK; 10 Hematology and Hematopoietic Transplant Unit, Azienda Ospedaliera di Rilievo Nazionale “San Giuseppe Moscati” (A.O.R.N. Giuseppe Moscati), Avellino, Italy; 11 Department of Haematology, Sheffield Teaching Hospitals NHS Foundation Trust, Sheffield, UK; 12 Clinical Hematology Department, Institut Català d’Oncologia-Hospitalet, Institut d’Investigació Biomèdica de Bellvitge (IDIBELL), University of Barcelona, Barcelona, Spain

**Keywords:** Leukaemia, Myelodysplastic syndrome

## Abstract

We looked at treatment rates and center density across countries for patients treated in 2022; 46,143 HCTs (19,011 (41.2%) allogeneic, 27,132 (58.8%) autologous) reported by 689 centers. 4329 patients received advanced cellular therapies, 3205 were CAR-T. We found considerable differences in utilization of autologous, allogeneic HCT and more so for CAR-T. Differences in procedure type and for allogeneic HCT in donor use and disease indication are highlighted. For instance, countries with the highest use of unrelated donors per 10 million inhabitants were Germany (297) and the Netherlands (230), for identical sibling HCT it was Israel (148) and Lebanon (113), for haploidentical it was Israel (94) and Italy (94) and for cord blood it was the Netherlands (24) and the United Kingdom (15). We looked at HCT use for specific indications in allogeneic HCT (AML CR1, MDS, MPN and BMF). We correlated treatment rates with GNI and with demographic age structure and show correlations in HCT and CAR-T use and center density, highest in Italy for allogeneic and autologous HCT and in Switzerland for CAR-T. Resource restricted countries tend to concentrate HCT use in a limited number of centers. These data are useful for comparisons across countries.

## Introduction

The European Society for Blood and Marrow Transplantation (EBMT) publishes an annual survey describing activity in hematopoietic cell transplantation (HCT) centers in Europe, including patients receiving autologous and allogeneic HCT and other cellular therapies. The survey is designed in the form of a single page spreadsheet [1].

HCT is an established procedure for many acquired or inherited disorders of the hematopoietic system, benign or neoplastic, including those of the immune system, and to facilitate enzyme replacement in metabolic disorders [2–4]. The activity survey of the EBMT, describing the status of HCT, has become an instrument to observe trends and monitor changes in HCT technology in Europe and associated countries [5–15]. The survey, using a standardized structure, captures the numbers of HCT from highly committed participating centers, stratified by indication, donor type and stem cell source over time [16–20] in a highly regulated environment. https://www.ema.europa.eu/en/documents/scientific-guideline/qualification-opinion-cellular-therapy-module-european-society-blood-marrow-transplantation-ebmt_en.pdf]. Cellular therapies qualify as medicinal products with hematopoietic cells for uses other than to replace the hematopoietic system [21–23]. Cellular therapies are part of the EBMT activity survey since 2018.

## Patients and methods

### Data collection and validation

We invited participating centers to report their data for 2022 using the activity survey as shown [20]. Patients receiving their first transplant in the survey year are reported by disease, donor type and stem cell source.

Quality control measures included several independent systems: confirmation of validity of data entered by the center, selective comparison of the survey data with MED-A data sets in the EBMT Registry database and crosschecking with National Registries.

### Participating Centers

The directory of HCT centers of members and non-members of the EBMT is updated annually according to the center’s current activity. In 2022, 731 centers from 54 countries were contacted (44 European and 10 associated countries); of which 689 centers responded. This corresponded to a 94.4% return rate and included 15.5% EBMT non-members. Forty-two active centers failed to report in 2022. The WHO regional office definitions were used to classify countries as European or non-European. Nine collaborating non-European countries participated in the 2022 survey: Algeria, Iran, Iraq, Lebanon, Nigeria, Saudi Arabia, South Africa, Tunisia, and United Arab Emirates. Their data, 2714 HCT in 2601 patients, from 28 actively transplanting centers made up 5.9% of the total data set and are included in all analyses. The population number for European countries in 2022 were obtained from Eurostats: (https://ec.europa.eu/eurostat) and the World Bank database for the non-European countries: (https://databank.worldbank.org). The following sources were used for economic data: gross national income (GNI): datacatalog.worldbank.org/; current health expenditure (CHE): who.int/data/gho/data/indicators/indicator-details/GHO/ and age structure: databank.worldbank.org/.

### Treatment rates

Treatment rates, defined as the total number of HCT or CAR-T treatment per 10 million inhabitants (10^7^) were computed, without adjusting for patients receiving their treatment in a foreign country or for refugee populations not considered inhabitants. Center density was defined as number of centers performing a specific type of treatment per country per 10 million inhabitants. Countries with a population greater than 2 million were included in the analysis.

### Analysis

In 2022, 19,011 allogeneic HCT, 27,132 autologous HCT and 3205 CAR-T treatment procedures were recorded [20]. Allogeneic HCT were from sibling donors (5084) unrelated donors (9913), haploidentical donors (3741), or cord blood donors (273). Wherever appropriate, treatment rates and center density are shown. The absolute numbers of treated patients are used for ranking centers by size shown in the supplemental data (Supplementary Fig. 1a–k). Correlations of treatment rates with wealth of a specific country using GNI were done by linear regression with or without logarithmic transformation where appropriate. *P* values and R^2^ is shown to exemplify the magnitude of the correlation. Similarly, correlations of treatment rates by age structure of a given country exemplified by percentage of persons above the age of 65 is shown. We looked at use of HCT technology, autologous and allogeneic, and among allogeneic particularly by donor use, i.e., sibling donor HCT, unrelated donor HCT, haploidentical donor HCT and cord blood donor HCT by country and by center. We selected allogeneic HCT indications e.g., AML in CR1, MDS, MPN and BMF to compare among countries. All centers reporting to the EBMT activity survey were included. For comparisons among countries, small countries with a population of less than 2 million were excluded. Center density was calculated as number of centers for a particular technology by 10 million inhabitants of a given country. Given the recent SARS-Cov2 pandemic we compared use of HCT and CAR-T technology in the year prior (2019) to and the first year post (2022) pandemic restricted to those centers with data reported in both years. We listed centers with high numbers of HCT or CAR-T procedures restricting these lists to the 25 centers with the highest activity to analyse differences among centers in technology use. In some plots the number is lower or slightly higher to accommodate countries with similar activity. For the correlation plots with economic and demographic data, all 46 countries with a population over 2 million reporting to the activity survey are included. Unfortunately, we do not have data on regulations in different countries to possibly explain differences in activity based on health care organization. Finally, we correlated use of unrelated donor HCT with availability of donors per country using data provided by the World Donor Marrow Association (WMDA Global Trends Report [2022].

## Results

### Transplantation and cell therapy frequencies and indications

Of the 689 centers reporting in 2022, 452 (65.6%) performed both allogeneic and autologous transplants; 222 (32.2%) restricted their activity to autologous HCT, and 11 (1.6%) to allogeneic transplants only. Four (0.6%) of the 689 responding centers reported no activity due to renovation or changes within the transplant unit. 463 centers reported having performed allogeneic HCT and 674 autologous HCT, these are considered separately within the center density analysis. In 2022, 3205 CAR-T treatments were reported by 214 centers in 28 countries. Almost all centers reporting CAR-T treatment reported autologous and allogeneic HCT as well. Among the 3205 patients receiving CAR-T products, 2259 (70.5%) were for lymphoma, 470 (14.7%) for myeloma, 380 (11.8%) for ALL and 96 (3%) for other diseases.

**Fig. 1:**
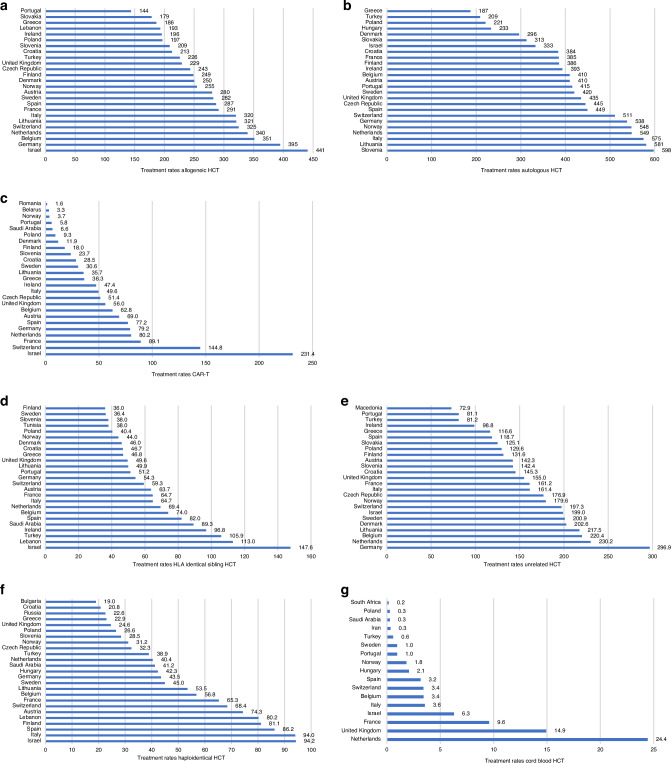

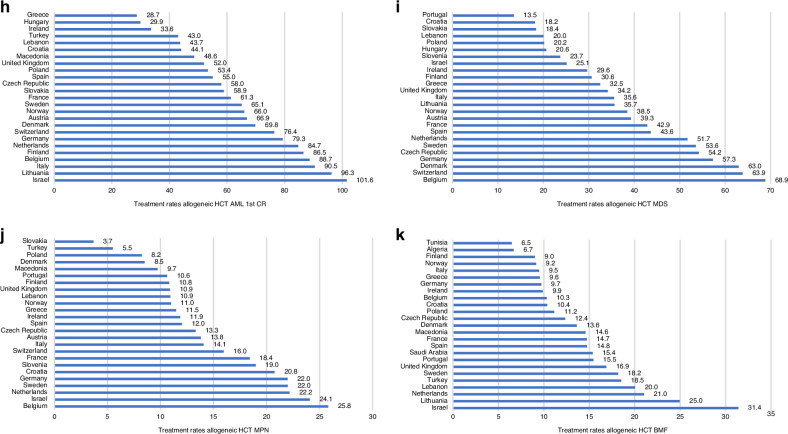
Treatment rate per 10 million inhabitants for the top 25 countries by treatment type; allogeneic and autologous HCT and CAR-T, by donor type and by four selected diseases in 2022. **a** Allogeneic HCT (all transplants). **b** Autologous HCT (all transplants). **c** CAR-T treatment (patients). **d** HLA identical sibling HCT (all transplants). **e** Unrelated HCT (all transplants). **f** Haploidentical family HCT (all transplants). **g** Cord blood HCT all donor types (all transplants). **h** Acute myeloid leukemia in 1st complete remission (1st transplant). **i** Myelodysplastic syndrome (1st transplant). **j** Myeloproliferative neoplasm (1st transplant). **k** Bone marrow failure (1st transplant).

Figure 1 shows treatment rates for allogeneic (Fig. 1a), autologous (Fig. 1b) HCT and CAR-T cell therapy (Fig. 1c) for the 25 countries with the highest activity in 2022. The treatment rate for allogeneic HCT ranges from 441 to 144 per 10^7^ inhabitants, for autologous HCT from 598 to 187 per 10^7^ inhabitants and for CAR-T from 231 to 1.6 per 10^7^ inhabitants showing greater variability among countries for CAR-T as compared to allogeneic and autologous HCT. The 3 countries with the highest rates of allogeneic HCT are Israel, Germany and Belgium, for autologous HCT these are Slovenia, Lithuania and Italy and for CAR-T these are Israel, Switzerland and France. Figure 1d–g shows treatment rates for allogeneic HCT by country and by donor type. For identical sibling HCT rates range from 148 to 36 per 10^7^ inhabitants, the countries with the 3 highest rates are Israel, Lebanon and Turkey (Fig. 1d). For unrelated donors (Fig. 1e) the range is from 297 to 73 per 10^7^ inhabitants, and the 3 countries with the highest rates are Germany, Netherlands and Belgium. Figure 1f shows the rates of haploidentical donor HCT, ranging from 94 to 19 per 10^7^ inhabitants, with the highest rates in Israel, Italy and Spain. For cord blood HCT rates vary from 24.4 to 0.2 per 10^7^ inhabitants, with the highest numbers in the Netherlands, United Kingdom and France (Fig. 1g).

Allogeneic transplant rates specific for 4 main disease entities analysed is shown in Fig. 1h–k. This includes AML in CR1, the frequency of which varies from 102 to 29 per 10^7^ inhabitants, and with Israel, Lithuania and Italy being the 3 countries with the highest rates (Fig. 1h). For MDS treatment rates varied from 69 to 13.5 per 10^7^ inhabitants, and Belgium, Switzerland and Denmark were the 3 countries with the highest rates (Fig. 1i). For MPN, rates per 10^7^ inhabitants varied from 26 to 4, with the highest rates observed in Belgium, Israel and the Netherlands (Fig. 1j). For bone marrow failure syndromes, rates varied from 31 to 6.5 per 10^7^ inhabitants and were the highest in Israel, Lithuania and the Netherlands. Of note, countries such as Lebanon, Turkey, Saudi Arabia, Algeria and Tunisia also featured among the top 25 countries for this indication (Fig. 1k).

Supplementary Fig. 1a–k, show a ranking of the 25 centers with the highest activity in allogeneic and autologous HCT and CAR-T (Supplementary Fig. 1a–c), for allogeneic HCT by donor type (Supplementary Fig.1d–g) and for allogeneic HCT per indication (Supplementary Fig. 1h–k). From these data it appears that countries with the highest transplantation rates do not necessarily provide the centers with the highest transplant activities. E.g., the center with the highest number of sibling HCT is Centre Pierre et Marie Curie in Algiers, in other countries availability of unrelated donors and economic power likely explains that Hamburg Eppendorf is the center with the largest number of unrelated donor HCT. The most active center with haploidentical HCT is the first state Pavlov medical university of St. Petersburg, for cord blood transplantation it is central Manchester NHS trust.

### Center density, gross national income and current health expenditure

**Fig. 2:**
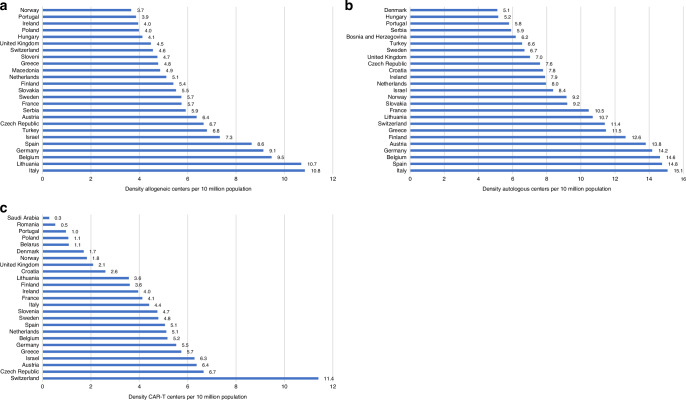
Center density per 10 million inhabitants for top 25 countries for allogeneic centers, autologous centers and CAR-T centers in 2022. **a** Centers performing allogeneic HCT. **b** Centers performing autologous HCT. **c** Centers performing CAR-T treatments.

In Fig. 2 we depicted center density by country, defined as the number of centers performing a specific type of treatment per 10 million inhabitants, again for the 25 countries with the highest density in 2022. Center density for allogeneic HCT was 10.8 to 3.7 centers per 10^7^ inhabitants, with the highest density observed in Italy, Lithuania and Belgium (Fig. 2a). The density for centers reporting autologous HCT was 15.1 to 5.1 per 10^7^ inhabitants with Italy, Spain and Belgium having the highest density (Fig. 2b). Figure 2c shows CAR-T center density varying from 11.4 to 0.3 per 10^7^ inhabitants, with the highest density seen in Switzerland, Czech Republic and Austria.

**Fig. 3:**
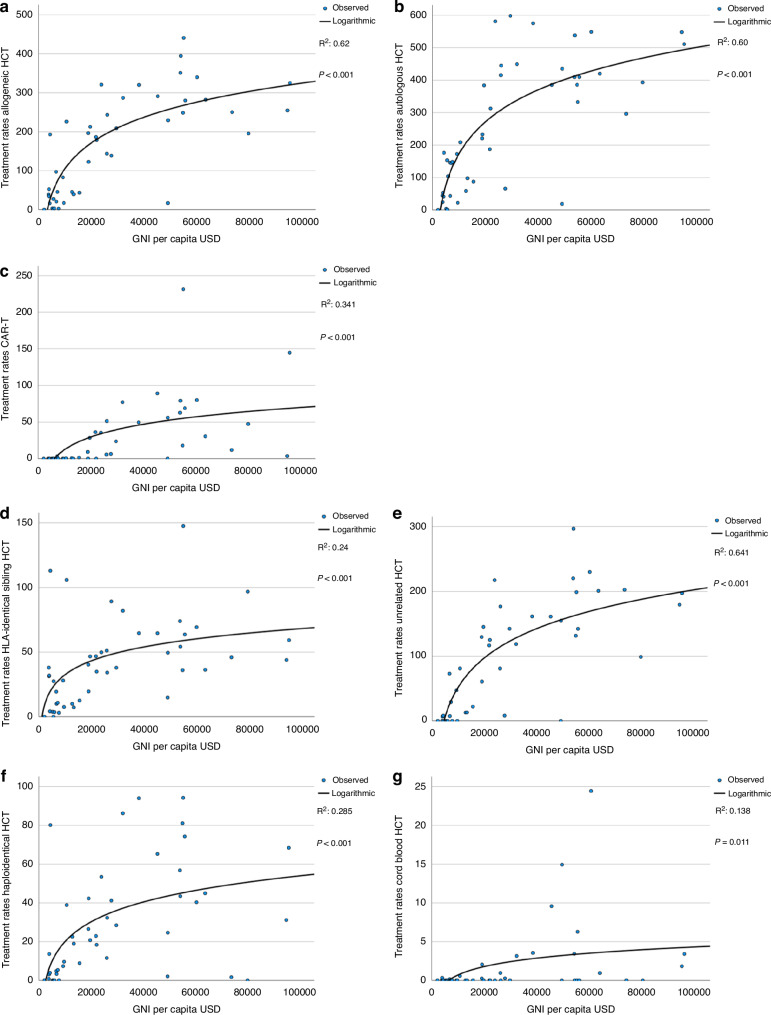

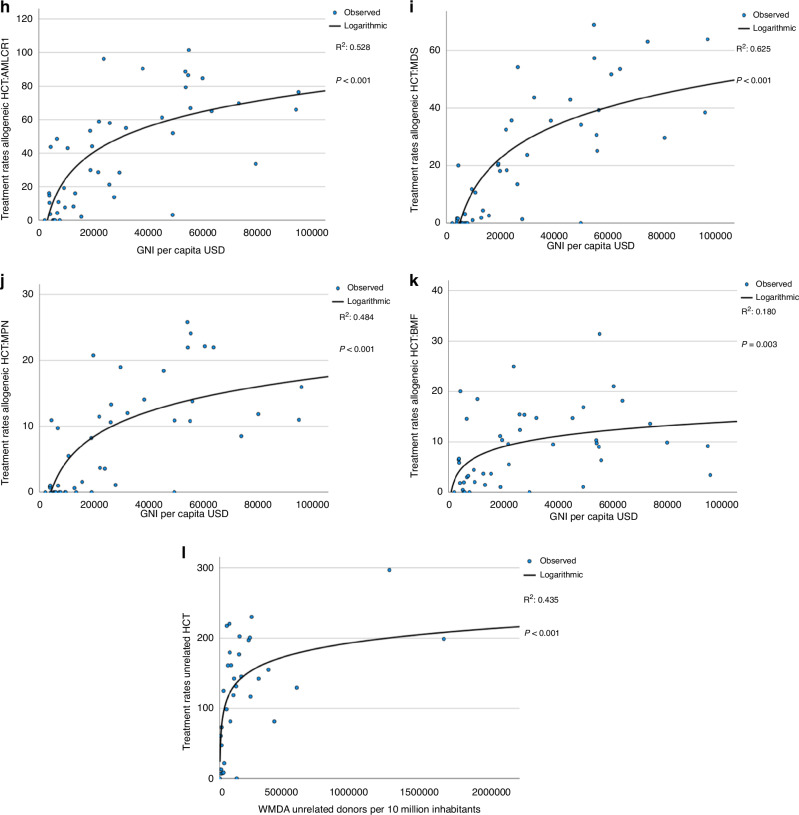
Logarithmic regression analysis for treatment rate per 10 million inhabitants by gross national income (GNI) per capita in USD in 46 countries with a population greater than 2 million in 2022. **a** Allogeneic HCT (all transplants). **b** Autologous HCT (all transplants). **c** CAR-T treatments (1st transplant). **d** HLA identical sibling HCT (all transplants). **e** Unrelated HCT (all transplants). **f** Haploidentical family HCT (all transplants). **g** Cord blood HCT (all transplants). **h** Acute myeloid leukemia in 1st complete remission (1st transplant). **i** Myelodysplastic syndrome (1st transplant). **j** Myeloproliferative neoplasm HCT (1st transplant). **k** Bone marrow failure HCT (1st transplant). **l** Unrelated donor HCT rate by availability of unrelated donors within WMDA registries.

Figure 3 shows logarithmic linear regression analyses of treatment rates by GNI per capita in USD. We included all 46 countries with a population greater than 2 million as opposed to the 25 countries with the highest HCT and cell therapy rates that were depicted in Fig. 1a–k. Data correlating treatment rates by current health expenditure (CHE) are not shown, as CHE was so highly correlated with GNI (R^2^ 0.95, p < 0.0001) that results were almost identical (Supplementary Fig. 2). GNI correlated significantly with allogeneic, autologous and CAR-T treatment rates. The fraction of the variability explained by the regression is (R^2^ 0.62, p < 0.001) for allogeneic HCT (Fig. 3a), (R^2^ 0.6, p < 0.001) for autologous HCT (Fig. 3b) and (R^2^ 0.34, p < 0.001) for CAR-T (Fig. 3c). Figure 3d–g shows the correlation of GNI with allogeneic donor types. Correlation of unrelated donor use with GNI (R^2^ 0.64, p < 0.001) (Fig. 3e) was stronger than for sibling donor (R^2^ 0.24, p < 0.001) (Fig. 3d) or for haploidentical donor (R^2^ 0.29, p < 0.001) (Fig. 3f) transplantation, as expected. The correlation between the use of cord blood and GNI (R^2^ 0.14, p = 0.011) (Fig. 3g) was weak, suggesting that the use of cord blood technology is driven by other factors. Figure 3l shows the correlation of the rate of unrelated donor transplants with donor availability through WMDA (R^2^ 0.43, p < 0.001). This correlation appeared moderately weak with several countries with high activity in unrelated donor HCT despite low numbers of available donors in the associated registry. In the supplementary section, Fig. 3 shows number of unrelated donors available per country per 10 million inhabitants. Similarly in Fig. 3h–k, transplant rates for AML in CR1 (R^2^ 0.53, p < 0.001) (Fig. 3h), MDS (R^2^ 0.63, p < 0.001) (Fig. 3i), and MPN (R^2^ 0.48, p < 0.001) (Fig. 3j), correlated strongly with GNI. Of note, transplant rates for BMF (R^2^ 0.18, p = 0.003) (Fig. 3k) demonstrated a much weaker correlation with GNI.

Supplementary Fig. 4a–k show correlations between the percentage of elderly in a country and use of various types of HCT and cell therapy procedures. As expected, allogeneic and autologous HCT rates increased with the age of the population (Supplementary Fig. 4a–c). Among allogeneic HCT, the use of unrelated donors correlated better with higher population age than the use of sibling or haploidentical donors (Supplementary Fig. 4d–g). When analysed per indication, it was observed that the rate of allogeneic transplants for AML CR1, MDS, and MPN were higher in older populations, but this was not the case for BMF as allogeneic HCT indication (Supplementary Fig. 4h–k).

**Fig. 4:**
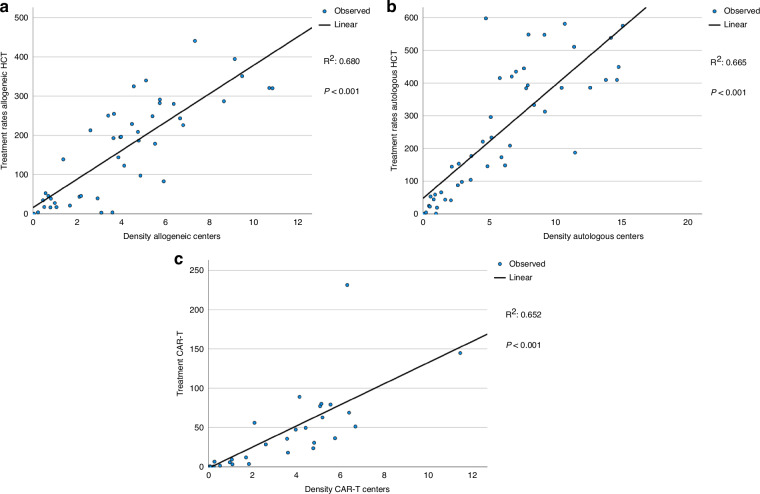
Linear regression analysis for treatment rate per 10 million inhabitants for transplant type by center density in 46 countries with a population greater than 2 million in 2022. **a** Allogeneic HCT (all transplants). **b** Autologous HCT (all transplants). **c** CAR-T treatment (patients).

**Fig. 5:**
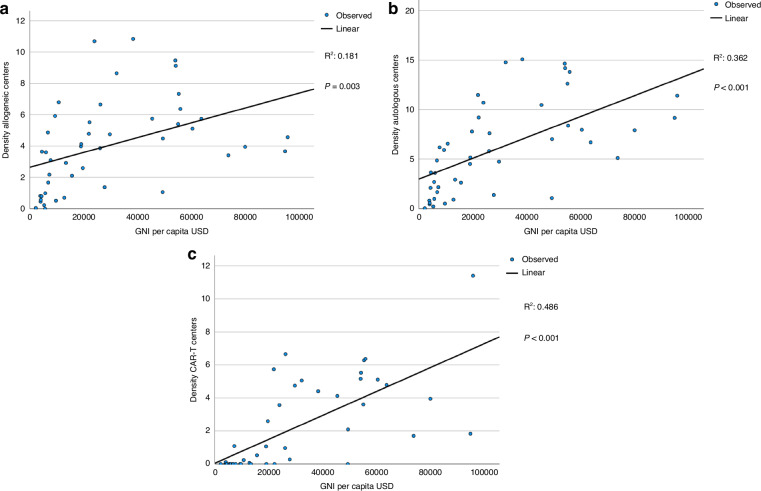
Linear regression analysis for center density by gross national income (GNI) per capita in USD in 46 countries with a population greater than 2 million in 2022. **a** Centers performing allogeneic HCT. **b** Centers performing autologous HCT. **c** Centers performing CAR-T treatments.

We then investigated whether center density was related to GNI. As expected, transplant rates for allogeneic HCT (R^2^ 0.68, p < 0.001) (Fig. 4a), autologous HCT (R^2^ 0.67, p < 0.001) (Fig. 4b) and for CAR-T (R^2^ 0.65, p < 0.001) (Fig. 4c) treatment correlated strongly with density of centers per country. Center density correlated with GNI as well, but this correlation was stronger for CAR-T center density than for HCT center density (Fig. 5a–c).

### SARS-CoV-2 and center activities

Table 1 shows the effect of the SARS-Cov2 pandemic focusing on activity in the 656 centers reporting to the activity survey in both 2019 and 2022. Overall, there is a small decrease in allogeneic HCT by 1.67% and a slightly larger decrease by 3.16% in autologous HCT. The small decrease in allogeneic activity is limited to HLA identical sibling donor transplants and cord blood transplants. In contrast, CAR-T activity increased by 183% in the same period. Looking at specific indications, use of allogeneic HCT for AML CR1, MDS, MPN, and BMF increased. A decrease was observed in other indications, particularly in lymphoid malignancies where CAR-T treatment has become available (data available in [20]).

**Table 1:** Difference in activity reported the year 2019, pre-SARS-Cov2 pandemic and 2022 post pandemic in 656 centers that reported in both years.

	2019	2022	Difference in absolute number of HCT 2022 -2019	% Difference 2022 -2019
All HCT (1st HCT plus additional HCT)				
Allogeneic HCT	18,855	18,540	−315	−1.67
Autologous HCT	27,058	26,204	−854	−3.16
Total HCT	45,913	44,744	−1169	−2.55
HLA identical sibling donor HCT including cord blood	5419	4860	−559	−10.32
Haploidentical family donor HCT including cord blood	3611	3630	19	0.53
Unrelated donor HCT including cord blood	9825	10,050	225	2.29
Cord blood HCT (all donor types)	304	273	−31	−10.20
1st HCT only (patient)				
Allogeneic HCT: AML CR1	3821	4088	267	6.99
Allogeneic HCT: MDS	2232	2251	19	0.85
Allogeneic HCT: MPN	781	839	58	7.43
Allogeneic HCT: BMF	966	1032	66	6.83
CAR-T (allogeneic plus autologous)	1127	3195	2068	183.50

*AML* acute myeloid leukemia, *MDS* myelodysplastic syndrome, *MPN* myeloproliferative neoplasm, *BMF* bone marrow failure.

## Discussion

The EBMT activity survey has been conducted annually since 1990 [1]. Over 46,000 transplants in almost 42,000 patients were reported in 2022. We observed a small decline in activity in autologous and allogeneic HCT when comparing treatment numbers before and after the Sars-COV2 pandemic (Table 1). The reason for this observed decline is unclear. Reasons may include availability of alternative therapies, competition within centers between transplant and CAR-T treatment, staff shortage and resource limitations after the pandemic ended or potential continued effects related to the pandemic. Whatever the reason, it did not affect the use of CAR-T cell therapy, increasing considerably during the same time.

The analysis presented here shows that differences among countries in use of transplant and CAR-T technologies can be related to economic and demographic factors. Treatment rates differed across European countries particularly the financially burdensome CAR-T technology. Some of the countries considered high users of allogeneic HCT and CAR-T technology, such as Israel and France, used considerably less autologous HCT. This is most likely explained by the fact that autologous HCT and CAR-T cell therapy are competitive technologies. As we have reported previously, the decrease in allogeneic and autologous HCT activity in lymphoid malignancies may be attributed to new therapeutic options including bispecific antibodies and CAR-T cells [21, 22]. Conversely, the number of autologous HCT for multiple myeloma continued to increase, despite the increased use of CAR T cell therapy for this indication [23]. This is probably related to the status of autologous HCT as first-line therapy in multiple myeloma, a status that is less affected by new developments such as CAR-T and bispecific antibody therapy, as these are reserved for more advanced disease stages.

Our data demonstrate that unrelated donor HCT is widely used in high income countries, whereas sibling donor transplantation is more frequent in countries with lower income. Haploidentical HCT was reported by high and middle income countries. Use of cord blood appeared to follow different rules, possibly related to the individualized importance attributed to this technology. Indications for allogeneic HCT varied depending on demographics of the population. Allogeneic HCT for AML, MDS, MPN was common in countries with a higher income and with higher proportions of persons greater than 65 years of age. Allogeneic HCT for BMF was used more commonly in countries with a younger population and appeared less dependent on national income. To our surprise, the correlation between donor availability in the WMDA registry and the use of unrelated donor HCT was not strong. This was explained by a few (high income) countries with a high use of unrelated donor HCT who themselves nevertheless had relatively low numbers of available donors in their registries.

Center density per country varied 3-fold for allogeneic and autologous HCT but more than 10-fold for CAR-T cell therapy for the top 25 countries. Not surprisingly, center density strongly correlated with treatment rates for allogeneic, autologous and CAR-T treatment. This may be interpreted as centers being created where required or as demand for activity being driven by center availability. Interestingly, center density did not seem strongly dependent on GNI. Especially for allogeneic HCT the correlation was weak and highly variable, particularly middle income countries. Countries with limited resources tend to concentrate the use of HCT technology in a limited number of centers. In addition, family size differs by region and income class [24, 25]. For example, the center with the highest number of sibling HCT is Centre Pierre et Marie Curie in Algiers. However, in other areas the availability of unrelated donors and economic power likely determine the size of a center. For example, Hamburg Eppendorf is the center with the largest number of unrelated donor HCT. Finally, we observed a relatively high allogeneic transplant activity for BMF in middle-income countries, exemplified by the list of large BMF centers being very different from the list of large MPN or MDS transplant centers. This may reflect either a higher incidence of BMF in middle-income countries, or a policy of centers in these countries to focus their efforts on serving specifically these patients.

In conclusion, we have used the EBMT survey to reflect significant international variation in the application of various HCT technology to disease indications. Central to the mission and vision of the EBMT is to promote access to appropriate care for all patients according to clinical need. Whilst this large international variation in activity and technology use may be explained by broad economic and epidemiological factors alongside team density and other models of HCT delivery and also access to competing modern non-HCT treatments across countries and their health services, future work is needed to comprehensively identify the reasons for such profound variation. Ultimately, survival outcomes in different disease indications, whether treated with HCT or non-HCT approaches (or both), are the most important consideration. The extension of registries and benchmarking to accommodate all elements of modern complex treatment pathways is crucial for a full understanding and correction of health inequalities [17, 26, 27].

## Supplementary information

Figures 1-4

## Data Availability

Datasets may be available upon request via EBMT Partnering (partnering@ebmt.org)
