# Supplementary material for: Utilization of hematopoietic cell transplantation and cellular therapy technology in Europe and associated Countries. Using the 2022 activity survey data to correlate with economic and demographic factors. A report from the EBMT
Source: Bone Marrow Transplant. 2024 Nov 22;60(2):227–36. doi: 10.1038/s41409-024-02459-0 (PMC11810786; doi:10.1038/s41409-024-02459-0)

Supplementary figure 1: absolute numbers of all allogeneic HCT (1a), all autologous HCT (1b) and CAR-T treatments (1c) for the 25 most active centers reporting in 2022.

Fig. 1a

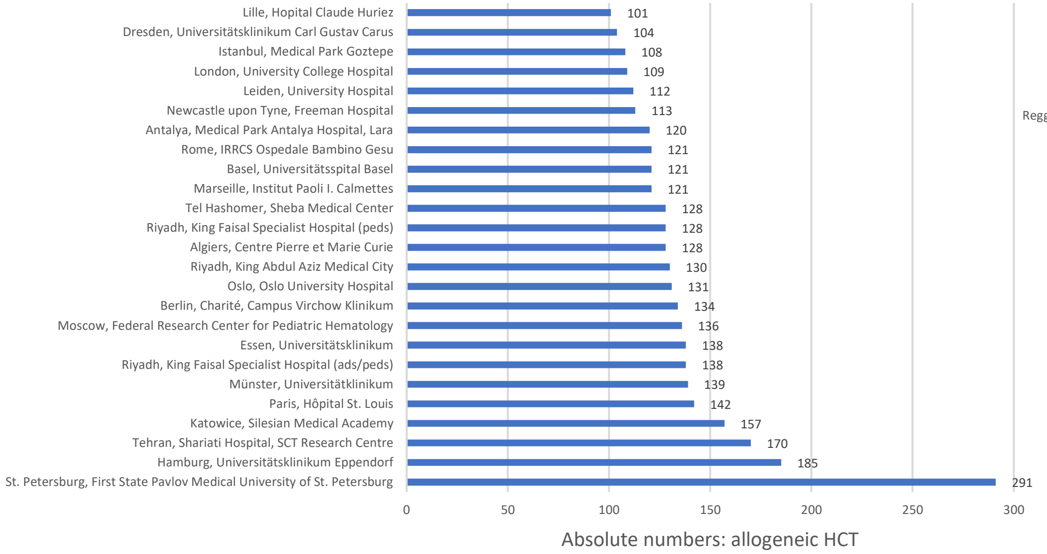

Fig.1b

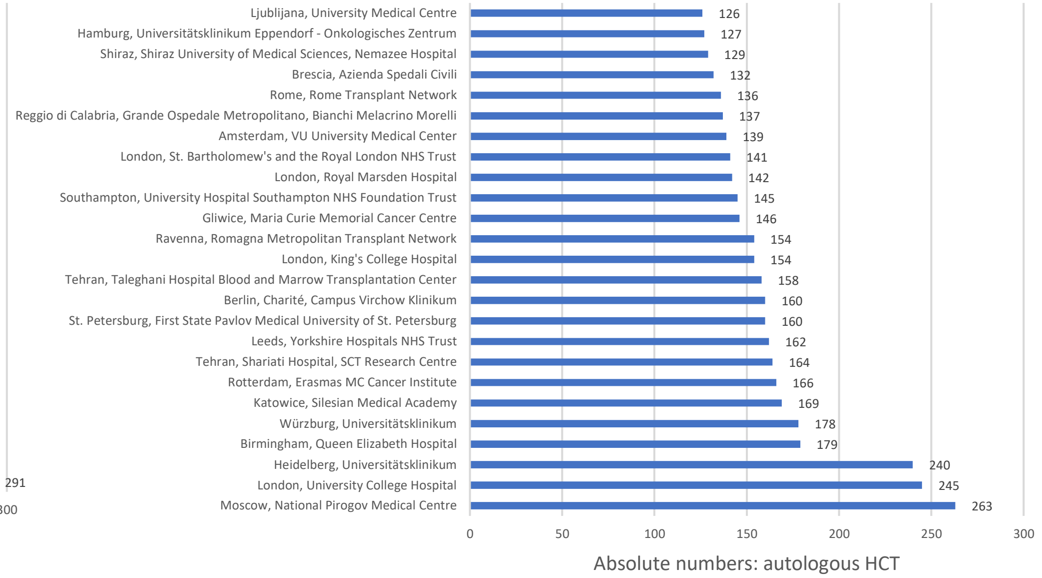

Fig. 1c

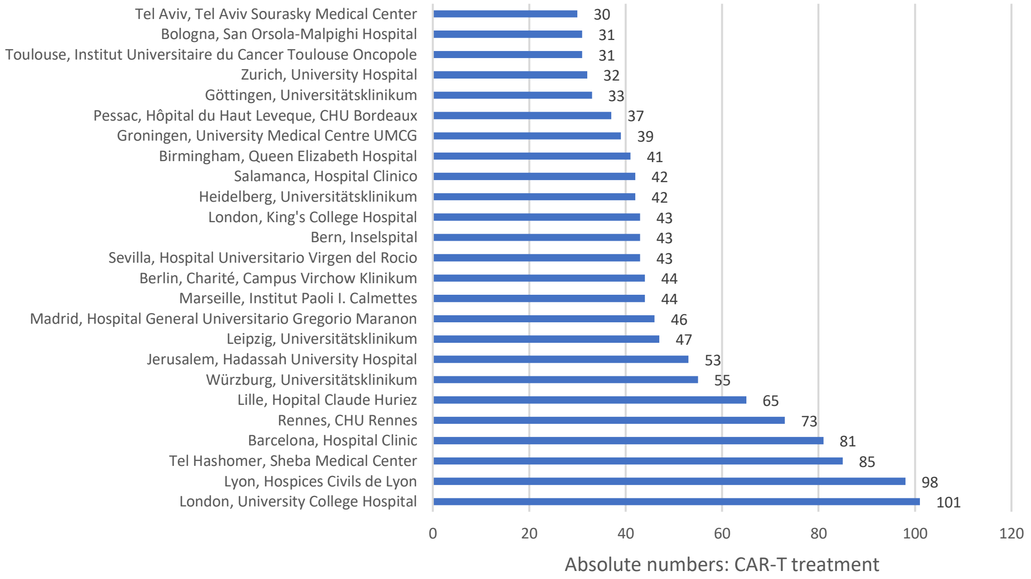

Supplementary figure 1defg: absolute numbers of all allogeneic HCT by donor type for the 25 most active centers reporting in 2022.

Fig. 1d

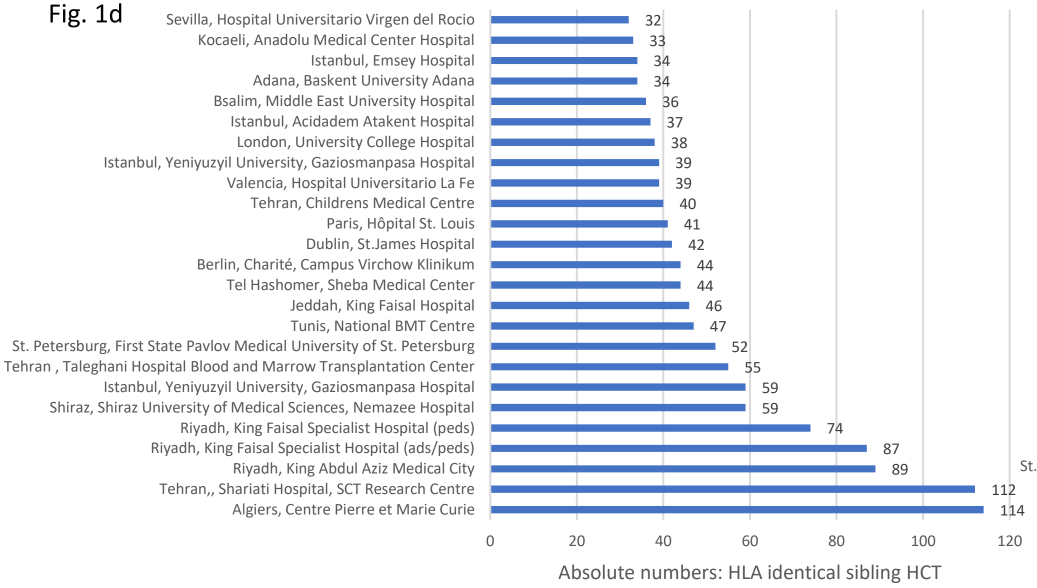

Fig. 1e

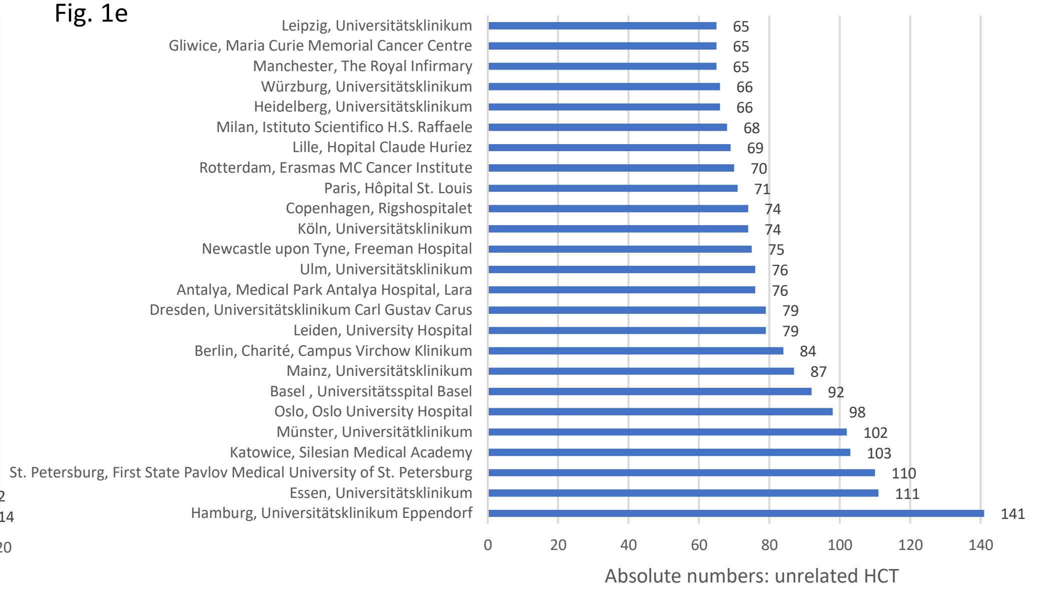

Fig. 1f

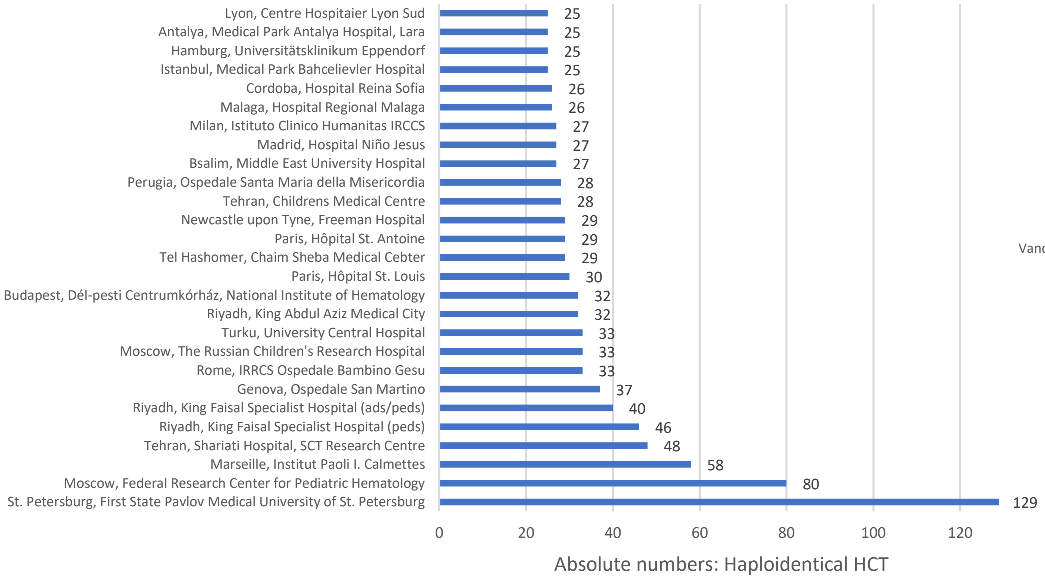

Fig. 1g

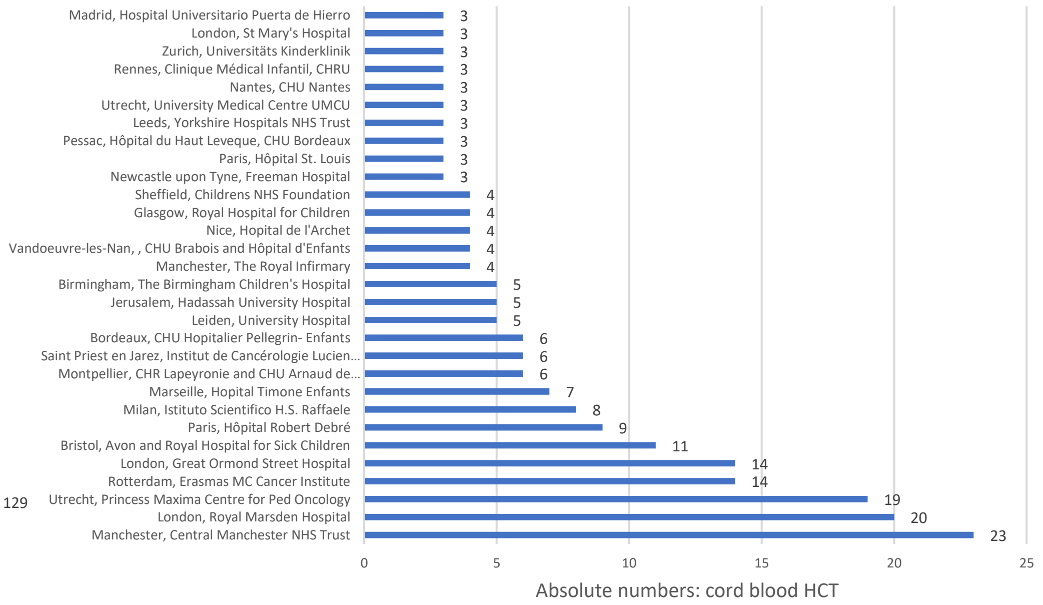

Supplementary figure 1hijk: absolute numbers of allogeneic 1st transplant by disease type for the 25 most active centers reporting in 2022.

Fig. 1h

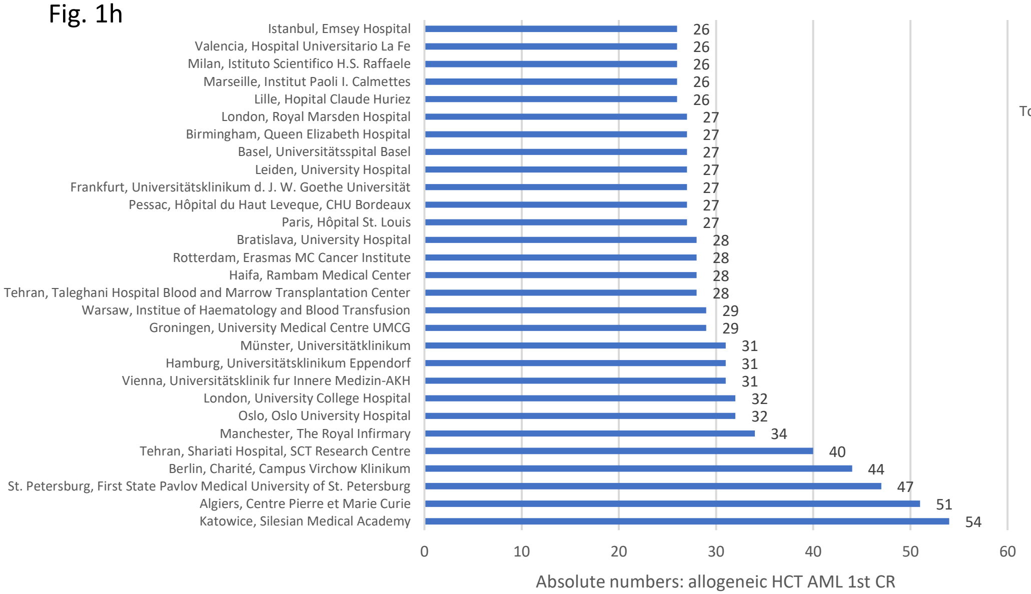

Fig. 1i

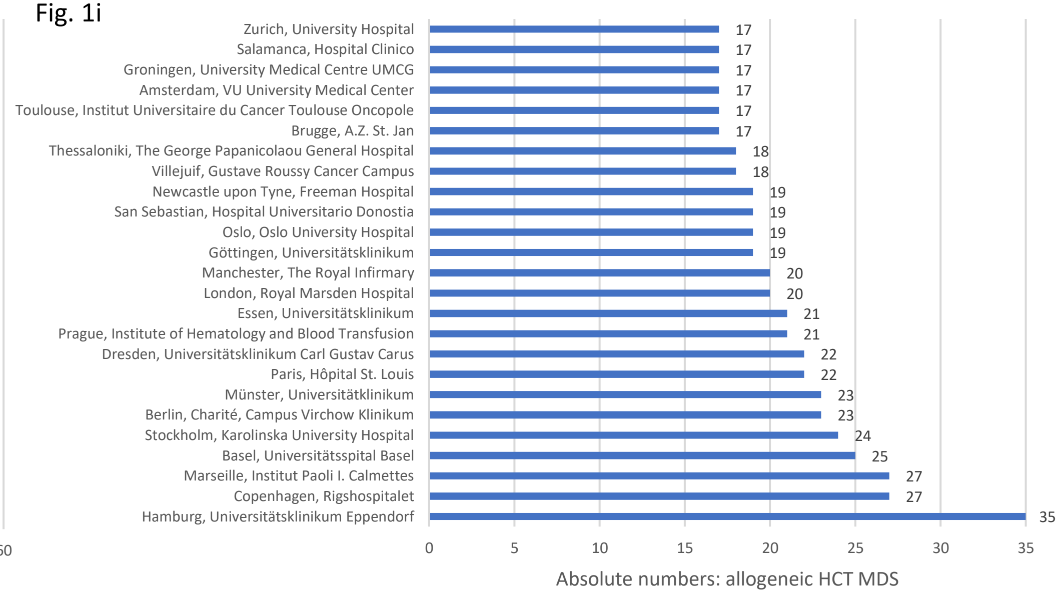

Fig. 1j

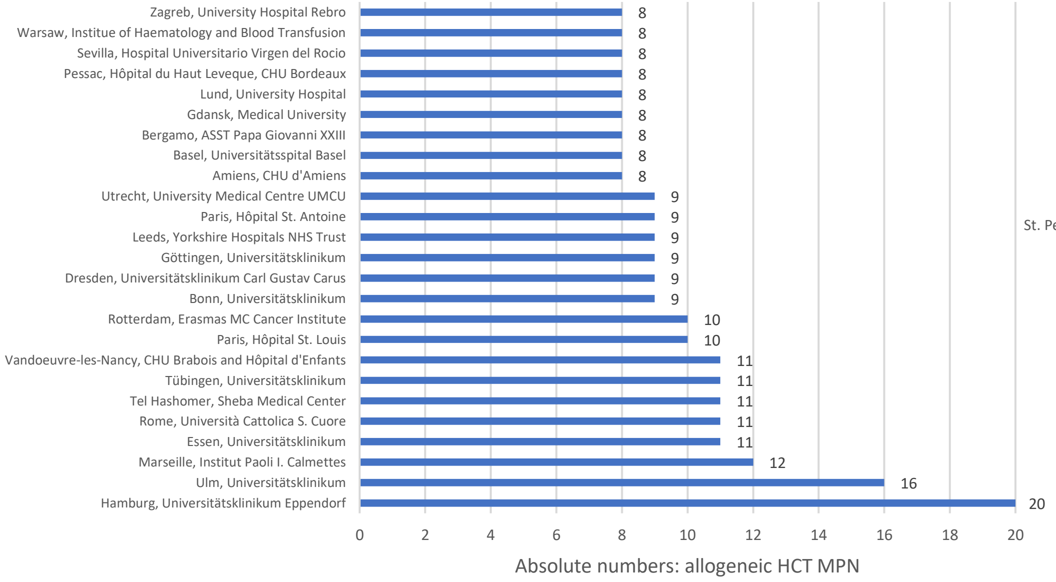

Fig. 1k

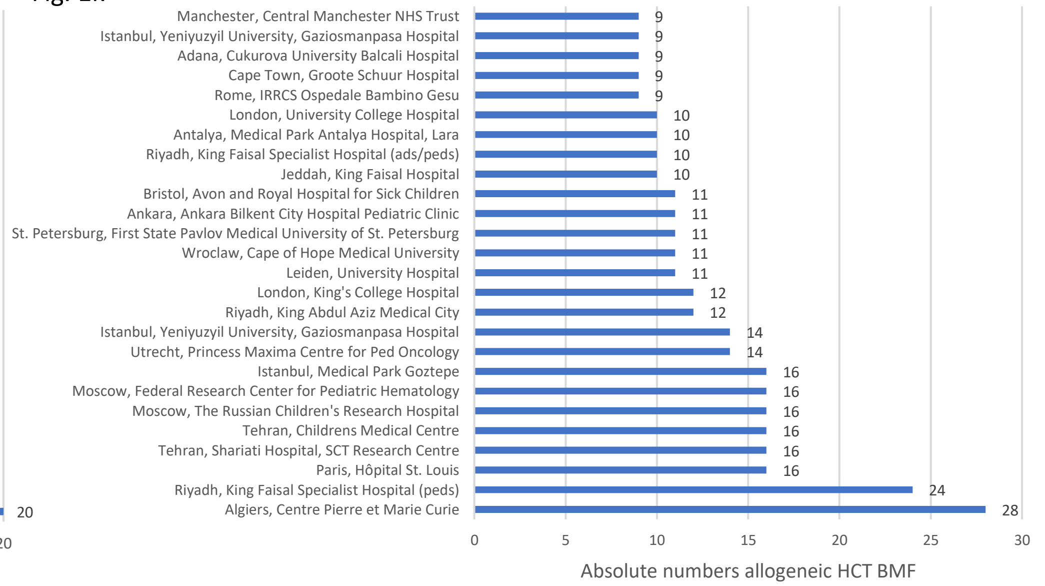

Supplementary figure 2: Linear regression analysis gross national income (GNI) by current healthcare expenditure (CHE) per capita in USD in 2022.

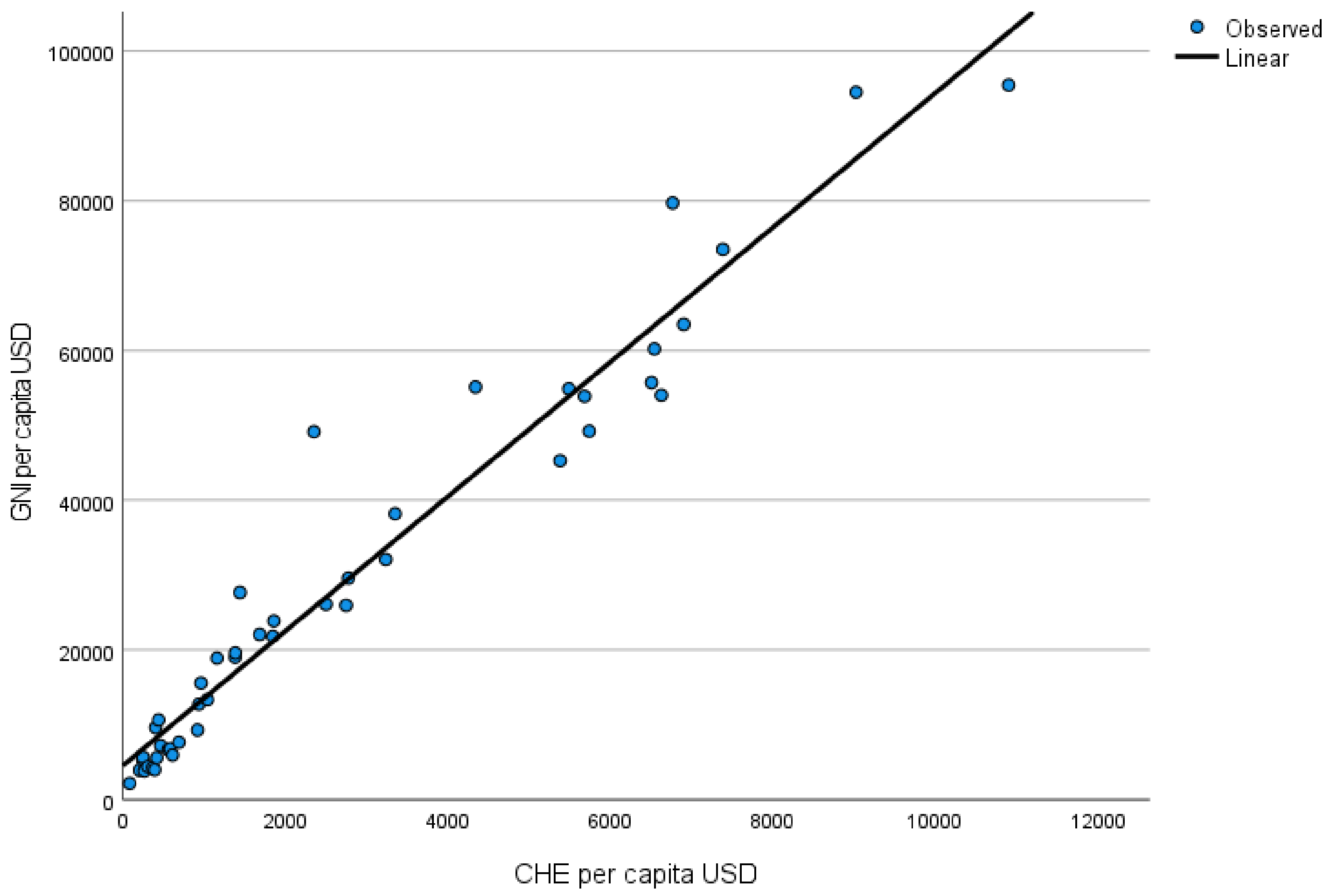

Supplementary figure 3: Number of unrelated donors per 10 million inhabitants by country in 2022 (WMDA Registry).

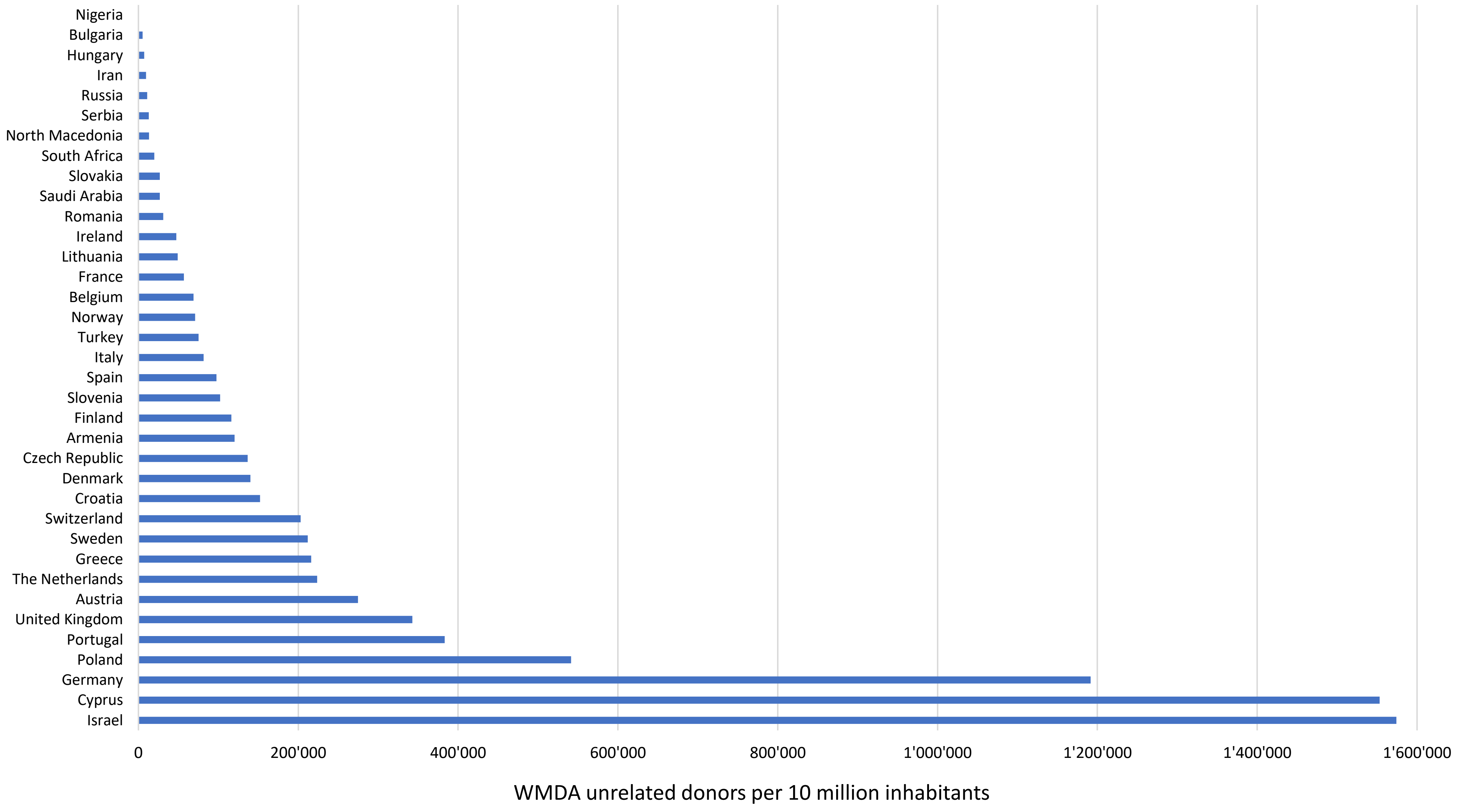

Supplementary figure 4abc: Linear regression analysis: treatment rate (TR) per 10 million inhabitants for treatment type by % population older than 65 years in 46 countries with a population greater than 2 million in 2022.

Fig. 4a

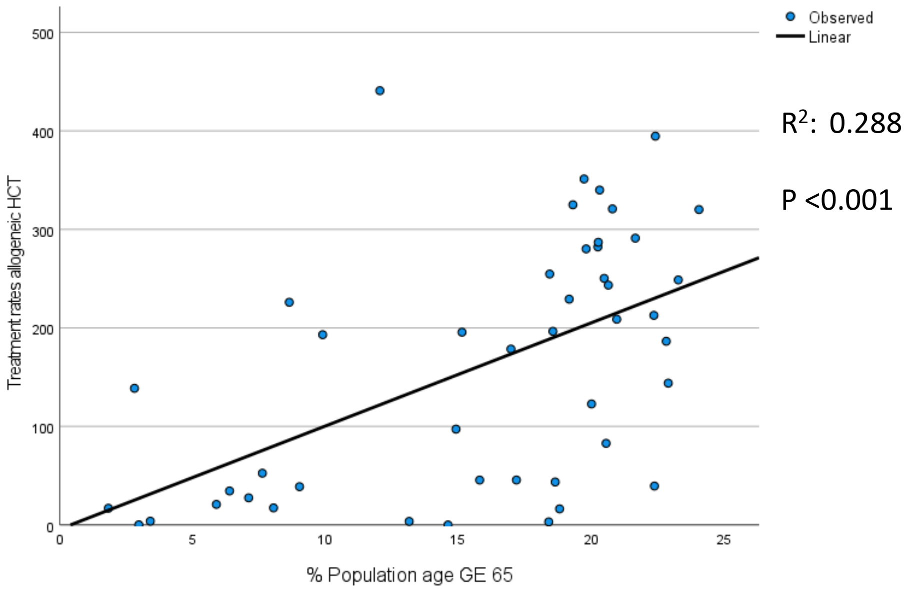

Fig. 4b

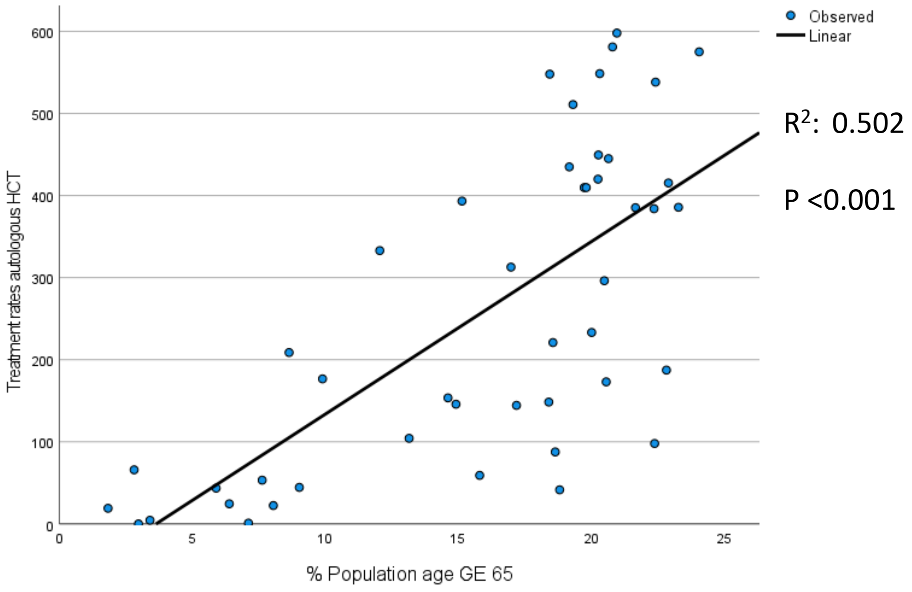

Fig. 4c

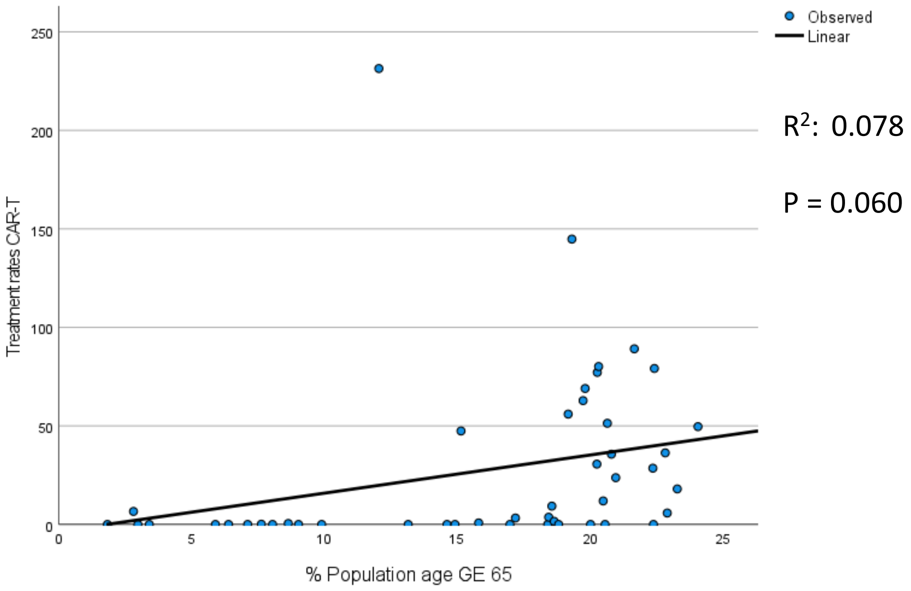

Supplementary figure 4defg: Linear regression analysis: treatment rate (TR) per 10 million inhabitants for donor type by % population older than 65 years in 46 countries with a population greater than 2 million in 2022.

Fig. 4d

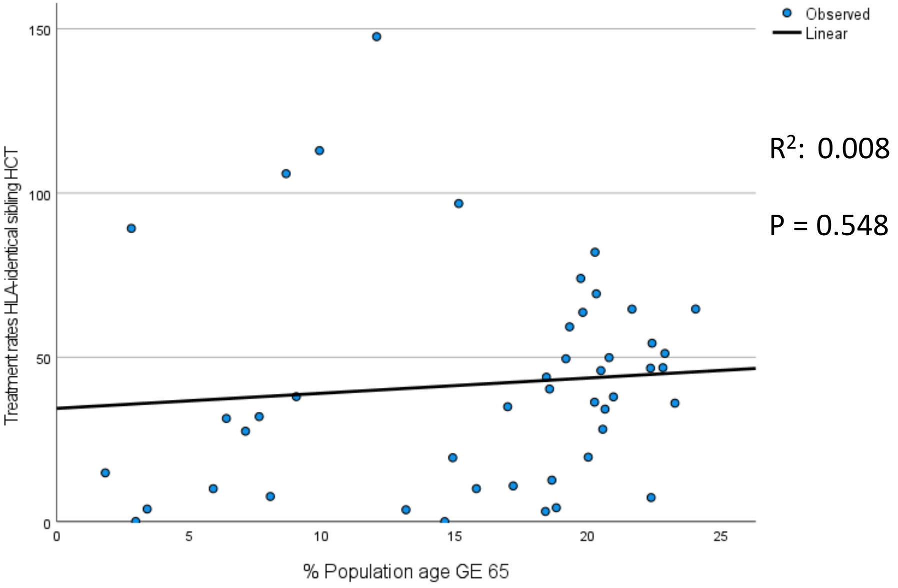

Fig. 4e

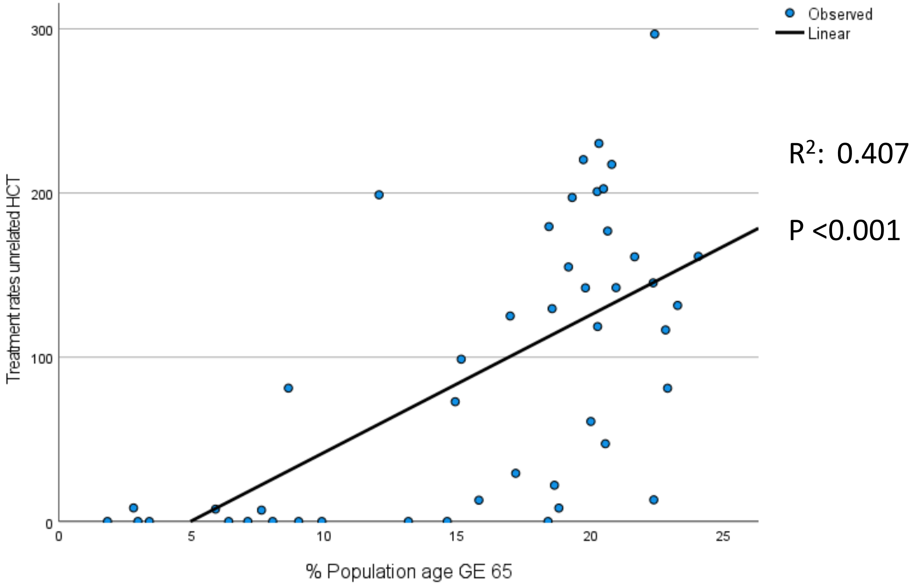

Fig. 4f

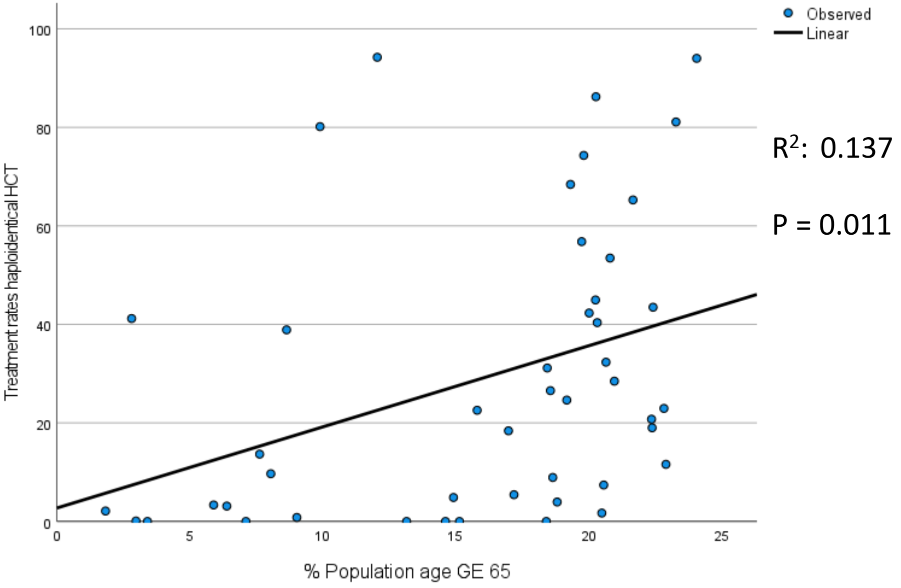

Fig. 4g

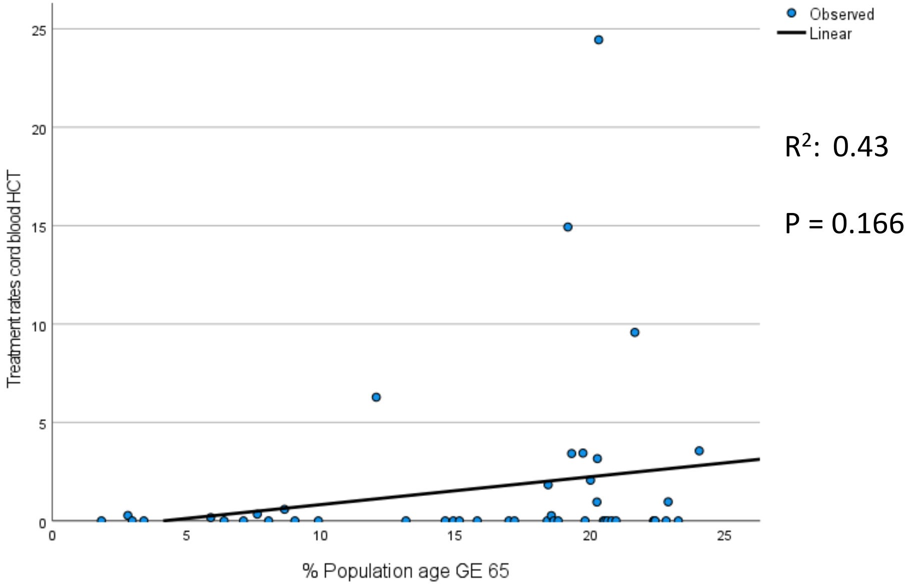

Supplementary figure 4hijk: Linear regression analysis: treatment rate (TR) per 10 million inhabitants in allogeneic 1st HCT by disease and % population older than 65 years in 46 countries with a population greater than 2 million in 2022.

Fig. 4h

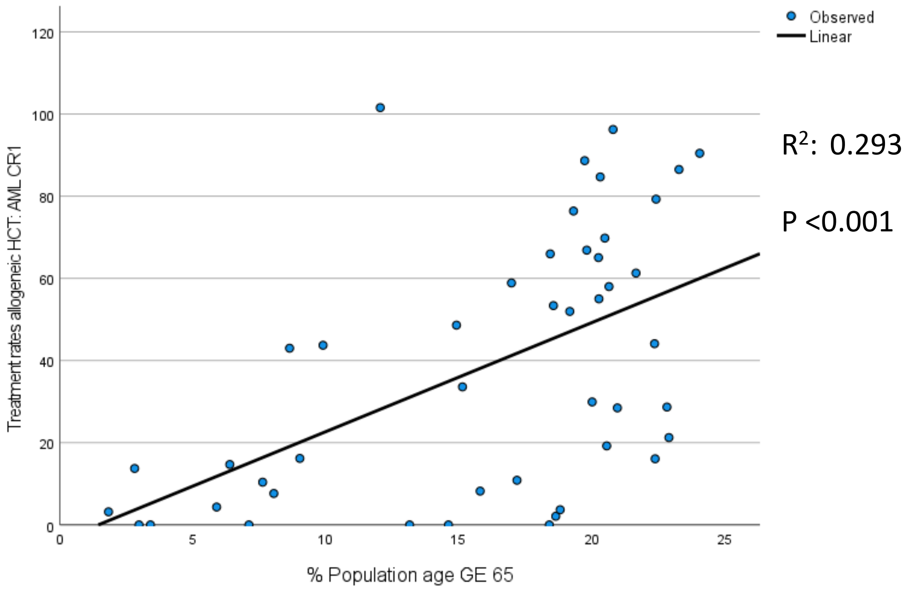

Fig. 4i

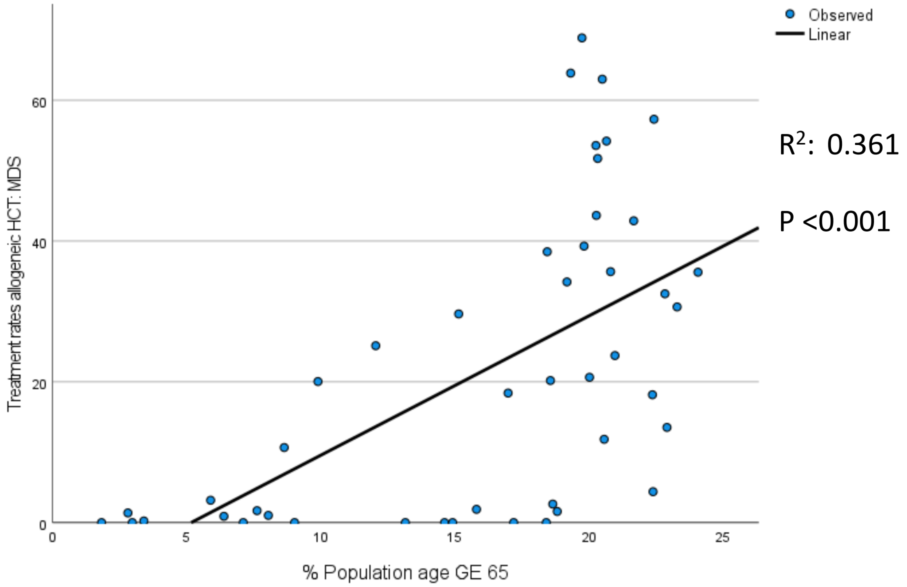

Fig. 4j

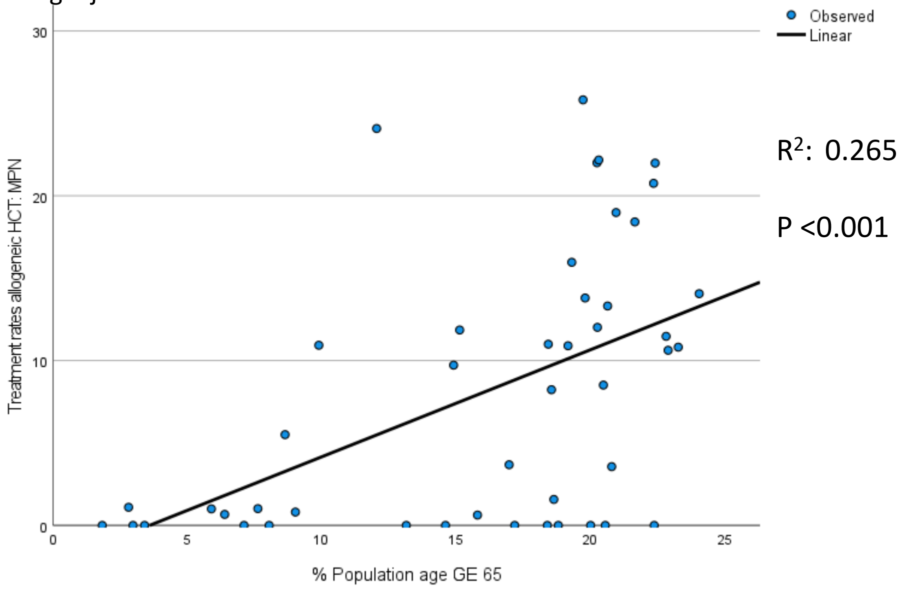

Fig. 4k

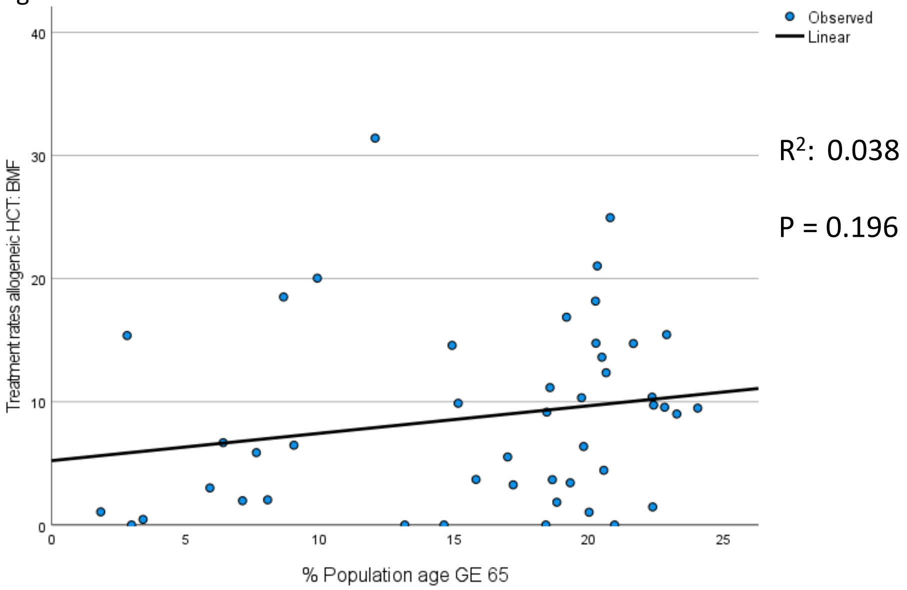

Supplement: Supplementary file 1 — Figures 1-4 [file 41409_2024_2459_MOESM1_ESM.pdf]
